# Natural Selection Constrains Neutral Diversity across A Wide Range of Species

**DOI:** 10.1371/journal.pbio.1002112

**Published:** 2015-04-10

**Authors:** Russell B. Corbett-Detig, Daniel L. Hartl, Timothy B. Sackton

**Affiliations:** 1 Department of Organismic and Evolutionary Biology, Harvard University, Cambridge Massachusetts, United States of America; 2 Department of Integrative Biology, University of California, Berkeley, Berkeley, California, United States of America; Institute of Science and Technology Austria (IST Austria), AUSTRIA

## Abstract

The neutral theory of molecular evolution predicts that the amount of neutral polymorphisms within a species will increase proportionally with the census population size (Nc). However, this prediction has not been borne out in practice: while the range of Nc spans many orders of magnitude, levels of genetic diversity within species fall in a comparatively narrow range. Although theoretical arguments have invoked the increased efficacy of natural selection in larger populations to explain this discrepancy, few direct empirical tests of this hypothesis have been conducted. In this work, we provide a direct test of this hypothesis using population genomic data from a wide range of taxonomically diverse species. To do this, we relied on the fact that the impact of natural selection on linked neutral diversity depends on the local recombinational environment. In regions of relatively low recombination, selected variants affect more neutral sites through linkage, and the resulting correlation between recombination and polymorphism allows a quantitative assessment of the magnitude of the impact of selection on linked neutral diversity. By comparing whole genome polymorphism data and genetic maps using a coalescent modeling framework, we estimate the degree to which natural selection reduces linked neutral diversity for 40 species of obligately sexual eukaryotes. We then show that the magnitude of the impact of natural selection is positively correlated with Nc, based on body size and species range as proxies for census population size. These results demonstrate that natural selection removes more variation at linked neutral sites in species with large Nc than those with small Nc and provides direct empirical evidence that natural selection constrains levels of neutral genetic diversity across many species. This implies that natural selection may provide an explanation for this longstanding paradox of population genetics.

## Abstract 2

Analysis of whole genome genetic variation data from 40 species shows that natural selection disproportionately depletes linked neutral variation in species with large population sizes, explaining why levels of neutral diversity do not scale with population size.

## Author Summary

A fundamental goal of population genetics is to understand why levels of genetic diversity vary among species and populations. Under the assumptions of the neutral model of molecular evolution, the amount of variation present in a population should be directly proportional to the size of the population. However, this prediction does not tally with real-life observations: levels of genetic diversity are found to be substantially more uniform, even among species with widely differing population sizes, than expected. Because natural selection—which removes genetically linked neutral variation—is more efficient in larger populations, selection on novel mutations offers a potential reconciliation of this paradox. In this work, we align and jointly analyze whole genome genetic variation data from a wide variety of species. Using this dataset and population genetic models of the impact of selection on neutral variation, we test the prediction that selection will disproportionally remove neutral variation in species with large population sizes. We show that genomic signature of natural selection is pervasive across most species, and that the amount of linked neutral variation removed by selection correlates with proxies for population size. We propose that pervasive natural selection constrains neutral diversity and provides an explanation for why neutral diversity does not scale as expected with population size.

## Introduction

The level of neutral genetic diversity within populations is a central parameter for understanding the demographic histories of populations [1], selective constraints [2], the molecular basis of adaptive evolution [3], genome-wide associations with disease [4], and conservation genetics [5]. Consequentially, numerous empirical surveys have sought to quantify the levels of neutral nucleotide diversity within species, and considerable theory has focused on understanding and predicting the distribution of genetic variation among species. All else being equal, under simple neutral models of evolution, levels of neutral genetic diversity within species are expected to increase proportionally with the number of breeding individuals (the census population size, Nc). Although this prediction is firmly established, surveys of levels of genetic variation across species have revealed little or no correlation between levels of genetic diversity and population size [6–9]. This discrepancy—first pointed out by Richard Lewontin in 1974 [6]—remains among the longest standing paradoxes of population genetics.

One possible explanation for this disagreement is an inverse correlation between mutation rate and population size. This is expected if there is relatively weak selection against alleles that cause higher mutation rates [8,10]. Alternatively, this paradox could result from greater impact in large populations of nonequilibrium demographic perturbations such as higher variance in reproductive success [11] or population size fluctuations [12]. Indeed, one recent empirical study suggests that demographic factors play an important role in shaping levels of genetic diversity within animal populations [13]. However, none of these potential explanations is sufficient to fully account for the observed patterns of neutral diversity across species [8].

Another potential cause of this paradox is the operation of natural selection on the genome [7,14,15]. Natural selection can impact levels of neutral diversity via the adaptive fixation of beneficial mutations (hitchhiking; HH) [7,15,16] and/or selection against deleterious mutations (background selection; BGS) [17,18]. Both processes purge neutral variants that are linked to selected mutations, implying that if natural selection is sufficiently common in the genome, it can reduce observed levels of neutral polymorphism. Furthermore, theoretical arguments [7,14,19] suggest that, when the impact of natural selection is substantial, the dependence of neutral diversity on population size is weak or even nonexistent. Although many authors have demonstrated that natural selection could, in principle, be sufficiently common to explain Lewontin’s paradox [7,8,14–16,20], few direct empirical tests of this explanation exist.

One unique prediction of the hypothesis that natural selection is a primary contributor to disparity between Nc and levels of neutral genetic variation within species is that natural selection will play a greater role in shaping the distribution of neutral genetic variation in species with large Nc. To test this prediction, we relied on the fact that the impact of natural selection on linked neutral diversity depends on the local recombinational environment. In regions of relatively low recombination, selected variants affect more neutral sites through linkage, and vice versa, in regions of relatively high recombination. The resulting correlation between recombination and polymorphism [21–26] (reviewed in depth in [27]) allows a quantitative assessment of the magnitude of the impact of selection on linked neutral diversity (e.g., [22,23,26,28]). Specifically, if the effects of linked selection can explain the lack of correlation between neutral diversity and population size, we expect that species with larger population sizes will display stronger correlations between recombination and polymorphism than those with smaller population sizes and show a concurrently larger impact of natural selection on levels of neutral diversity across the genome.

Although empirical studies that explore the relationship between neutral diversity and population size are relatively infrequent compared to theoretical studies on this topic, there are two interesting patterns that merit consideration here. First, the proportion of nonsynonymous substitutions that have been driven to fixation by positive selection varies widely across taxa. In humans [29], yeast [30], and many plant species [31], estimates of this proportion are close to zero. In contrast, in *Drosophila* [32,33], mice [34], and *Capsella grandiflora* [35], as well as other taxa (reviewed in [8]), a large fraction of nonsynonymous substitutions are inferred to have been driven to fixation by positive selection, implying that natural selection is common in the genomes of these organisms (which generally have large Nc). Second, the strength of the correlation between polymorphism and recombination varies widely among the limited number of taxa [8,27] that have been studied in depth. Here again, *Drosophila* [21,25,36] is among the taxa that shows the strongest correlation and thus the clearest evidence for natural selection, and the correlation in *Drosophila* is substantially larger than, for example, in humans [28].

In a related study to the work presented here, Bazin et al. [37] showed that there is no correlation between nucleotide diversity in nonrecombining mtDNA and nucleotide diversity in the nuclear genome. While this is consistent with some predictions of theoretical work on this subject, the mitochondrion has unusual patterns of replication and inheritance, and it is therefore challenging to disentangle the processes that generate diversity from those that shape its distribution across the genome. Although suggestive, the evidence accrued thus far is fragmentary, has not been analyzed in aggregate, and varies widely in quality of samples, data collection, and analyses performed [8,27]. It is therefore difficult to draw firm conclusions about the relative importance and prevalence of natural selection in shaping patterns of genetic variation in the genome based on existing studies.

Due to rapid advances in genome sequencing technologies, whole genome polymorphism data are now available for a wide variety of species (e.g., [36,38]), and these data enable us to conduct a quantitative test of the natural selection hypothesis as an explanation for Lewontin's paradox. Towards this, we identified 40 species with sufficiently high quality reference genomes, linkage maps, and polymorphism data to enable a broad-scale, robust comparison of the relative strength of correlation between polymorphism and recombination rate within a single unified alignment, assembly, and analysis framework. Using these data, and reasonable proxies for Nc, we show that the effect of selection on linked nucleotide diversity is indeed strongly correlated with population size. In other words, natural selection plays a disproportionately large role in shaping patterns of genetic variation in species with large Nc, confirming the idea that natural selection is an important contributor to Lewontin’s paradox.

## Results

### Genomic Datasets and Modeling Approach

We identified 40 species (15 plants, 6 insects, 2 nematodes, 3 birds, 5 fishes, and 9 mammals) for which a high-quality reference genome, a high-density, pedigree-based linkage map, and genome-wide resequencing data from at least two unrelated chromosomes within a population were available (Table 1, S1 Table, S2 Table). Because our model (below) requires that recombination has been sufficiently frequent to uncouple genealogies across large tracts of DNA on chromosomes, we required that each species have an obligatory sexual portion of its life cycle. This requirement necessarily excludes clades such as bacteria, which are predominantly clonally propagated. Nonetheless, extending this framework to bacterial taxa will be an important step towards understanding the mechanisms by which natural selection shapes patterns of variation across the tree of life. Additionally, our sampling is biased towards more commonly studied clades (e.g., mammals), but this is unavoidable in this type of analysis, and there is no reason in principle why this taxonomic bias would affect the basic conclusions we describe here, as the sampled taxa likely span a large range of census population sizes.

**Table 1:** List of species used in this work.

Species	Common Name	Kingdom	Subgroup
*Anopheles gambiae*	African malaria mosquito	Animalia	Invertebrate
*Apis mellifera scutellata*	Honeybee	Animalia	Invertebrate
*Arabidopsis thaliana*	Thale cress	Plantae	Herbaceous
*Bombyx mandarina*	Silkworm	Animalia	Invertebrate
*Bos taurus*	Cow	Animalia	Vertebrate
*Brachypodium distachyon*	Purple false brome	Plantae	Herbaceous
*Caenorhabditis briggsae*	Roundworm	Animalia	Invertebrate
*Caenorhabditis elegans*	Roundworm	Animalia	Invertebrate
*Canis lupus*	Wolf	Animalia	Vertebrate
*Capsella rubella*	Pink Shepherd's Purse	Plantae	Herbaceous
*Citrullus lanatus lanatus*	Watermellon	Plantae	Herbaceous
*Citrus reticulata*	Mandarin Orange	Plantae	Woody
*Cucumis sativus var*. *hardwickii*	Cucumber	Plantae	Herbaceous
*Cynoglossus semilaevis*	Tongue sole	Animalia	Vertebrate
*Danio rerio*	Zebrafish	Animalia	Vertebrate
*Drosophila melanogaster*	Fruitfly	Animalia	Invertebrate
*Drosophila pseudoobscura*	Fruitfly	Animalia	Invertebrate
*Equus ferus przewalskii*	Prewalksii's horse	Animalia	Vertebrate
*Ficedula albicollis*	Collared flycatcher	Animalia	Vertebrate
*Gallus gallus*	Chicken	Animalia	Vertebrate
*Gasterosteus aculeatus*	Stickleback	Animalia	Vertebrate
*Glycine soja*	Wild soybean	Plantae	Herbaceous
*Gossypium raimondii*	New world cotton	Plantae	Woody
*Heliconius melpomene melpomene*	Postman butterfly	Animalia	Invertebrate
*Homo sapiens*	Human	Animalia	Vertebrate
*Lepisosteus oculatus*	Spotted gar	Animalia	Vertebrate
*Macaca mulatta*	Rhesus macaque	Animalia	Vertebrate
*Medicago truncatula*	Barrel medic	Plantae	Herbaceous
*Meleagris gallopavo*	Turkey	Animalia	Vertebrate
*Mus musculus castaneus*	House mouse	Animalia	Vertebrate
*Oryza rufipogon*	Wild rice	Plantae	Herbaceous
*Oryzias latipes*	Medaka	Animalia	Vertebrate
*Ovis canadensis*	Bighorn sheep	Animalia	Vertebrate
*Papio anubis*	Olive baboon	Animalia	Vertebrate
*Populus trichocarpa*	Black cottonwood	Plantae	Woody
*Prunus davidiana*	David's peach	Plantae	Woody
*Setaria italica*	Foxtail millet	Plantae	Herbaceous
*Sorghum bicolor ssp*. *verticilliflorum*	Wild Sudan grass	Plantae	Herbaceous
*Sus scrofa*	Wild boar	Animalia	Vertebrate
*Zea mays ssp*. *parviglumis*	Teosinte	Plantae	Herbaceous

After acquiring sequence data, we developed and implemented a bioinformatic pipeline to align, curate, and call genotype data for each species (see S1 Fig and methods for a full description of the bioinformatics pipeline). We further used the available genetic maps to estimate recombination rates across the genomes. Across all species, we analyzed recombination across nearly 385,000 markers and aligned more than 63,000,000,000 short reads. This is therefore one of the largest comparative population genomics dataset that has been assembled to date.

We used both simple nonparametric correlations and explicit coalescent models to test for a relationship between the impact of selection on linked neutral diversity and census size. Although correlations between recombination rate and neutral diversity are informative, the extensive literature in theoretical population genetics provides an opportunity to develop a robust modeling approach. Two primary types of selection can introduce a correlation between recombination rate and levels of nucleotide diversity: background selection (BGS) and hitchhiking (HH). Here, we are not primarily concerned with distinguishing between the two models, and so focus on their joint effects. In addition to combining BGS and HH, we would also like to relax the assumption that these processes act uniformly across the genome. All else being equal, regions of the genome with a higher density of potential targets of selection should experience a greater reduction in neutral diversity.

**Fig 1:**
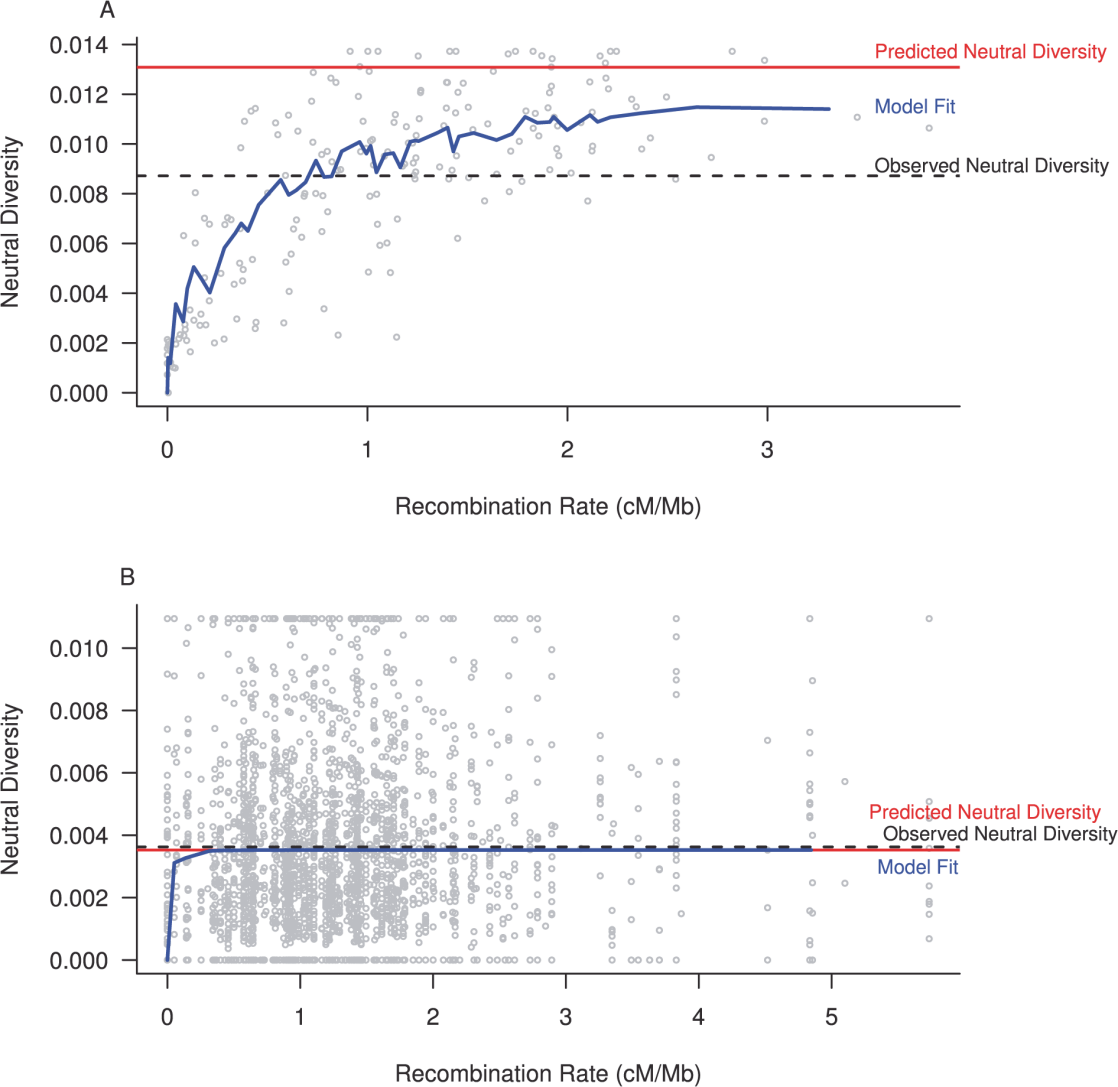
Estimating the impact of selection on linked neutral variation. To obtain a direct estimate of the amount of linked neutral variation removed by selection, we fit a population genetic model incorporating HH and BGS effects to the estimates of neutral diversity and recombination rate in 500 kb windows across the genome. Model fit (blue), predicted neutral diversity in the absence of selection (red), and observed genetic diversity (dashed) are shown for a species with large population size (*Drosophila melanogaster*, part A) and small population size (*Equus ferus przewalskii*, part B). The magnitude of the impact of selection on linked neutral diversity is estimated as 1 – (observed neutral diversity / neutral diversity in the absence of selection). R code to replicate this figure is available at https://github.com/tsackton/linked-selection/blob/master/final_analysis/figure1.R.

Starting from considerable prior theoretical work [14,17,18,32,39–41], we develop an explicit model relating polymorphism, recombination rate, and density of functional elements in the genome. We fit both a joint model that allows for both HH and BGS, as well as models of BGS only, HH only, and a purely neutral model (in which there is no predicted correlation between recombination or functional density and neutral diversity). Using these models, we estimate the proportion of neutral diversity removed by linked selection for beneficial alleles and/or against deleterious alleles (Fig. 1) for each species, as well as the relative likelihood of each model.

In practice, it is not feasible to determine Nc for the majority of species we studied. Instead, we used the species’ geographic range and individual body size as proxies for Nc. Size has been previously validated as a proxy for individual density in a wide variety of taxa and ecosystems (e.g., [42–44]). Under some simplifying assumptions, the product of geographic range and local density should be sufficient to roughly estimate a species census population size, and each factor is expected to independently capture some information related to species’ Nc. Specifically, we expect that range will be positively correlated with Nc, size will be negatively correlated with Nc, and Nc will be positively correlated with the impact of selection.

### Natural Selection Removes More Linked Neutral Variation in Species with Large Census Sizes

For many of the species that we studied, it is clear that selection plays a central, even dominant, role in shaping patterns of neutral genetic diversity. Specifically, both our correlation analysis and our explicit modeling support the hypothesis that natural selection on linked sites eliminates disproportionately more neutral polymorphism in species with large Nc, and in this way, natural selection truncates the distribution of neutral genetic diversity.

**Fig 2:**
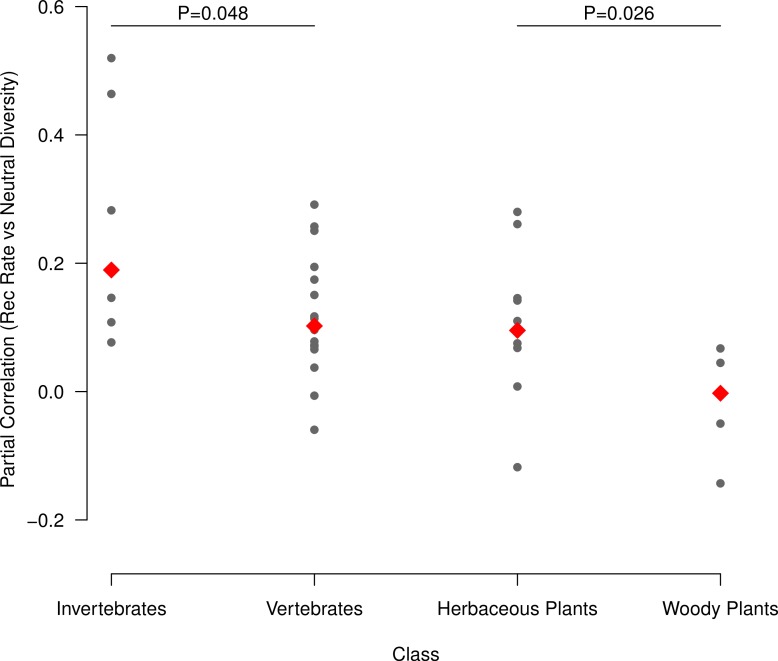
The correlation between neutral diversity and recombination rate is stronger in taxonomic classes expected to have larger population sizes. We computed partial correlations (Kendall's τ) between neutral diversity and recombination rate (estimated in 500 kb windows across the genome), accounting for variation in functional density (measured as proportion of sites in a window that are part of an annotated protein-coding exon). The significance of differences in median τ (red diamonds) between vertebrates and invertebrates, or between woody and herbaceous plants, is based on Wilcoxon Rank Sum Tests. Raw data underlying this figure can be found in S1 Data.

At a coarse scale, there is a stronger correlation between polymorphism and recombination in invertebrates (mean partial τ after correcting for gene density = 0.247), which likely have a large Nc on average, than in vertebrates (median partial τ = 0.118), which likely have a smaller Nc on average (two-tailed permutation *p* = 0.021). We observe similar patterns for herbaceous plants (mean partial τ = 0.106) versus woody plants (mean partial τ = −0.020; two-tailed permutation *p* = 0.058) and for medians as opposed to means (Fig. 2). When we repeat the analysis with alternate window sizes, we observe consistent effect sizes, albeit occasionally with reduced statistical support (S3 Table).

**Fig 3:**
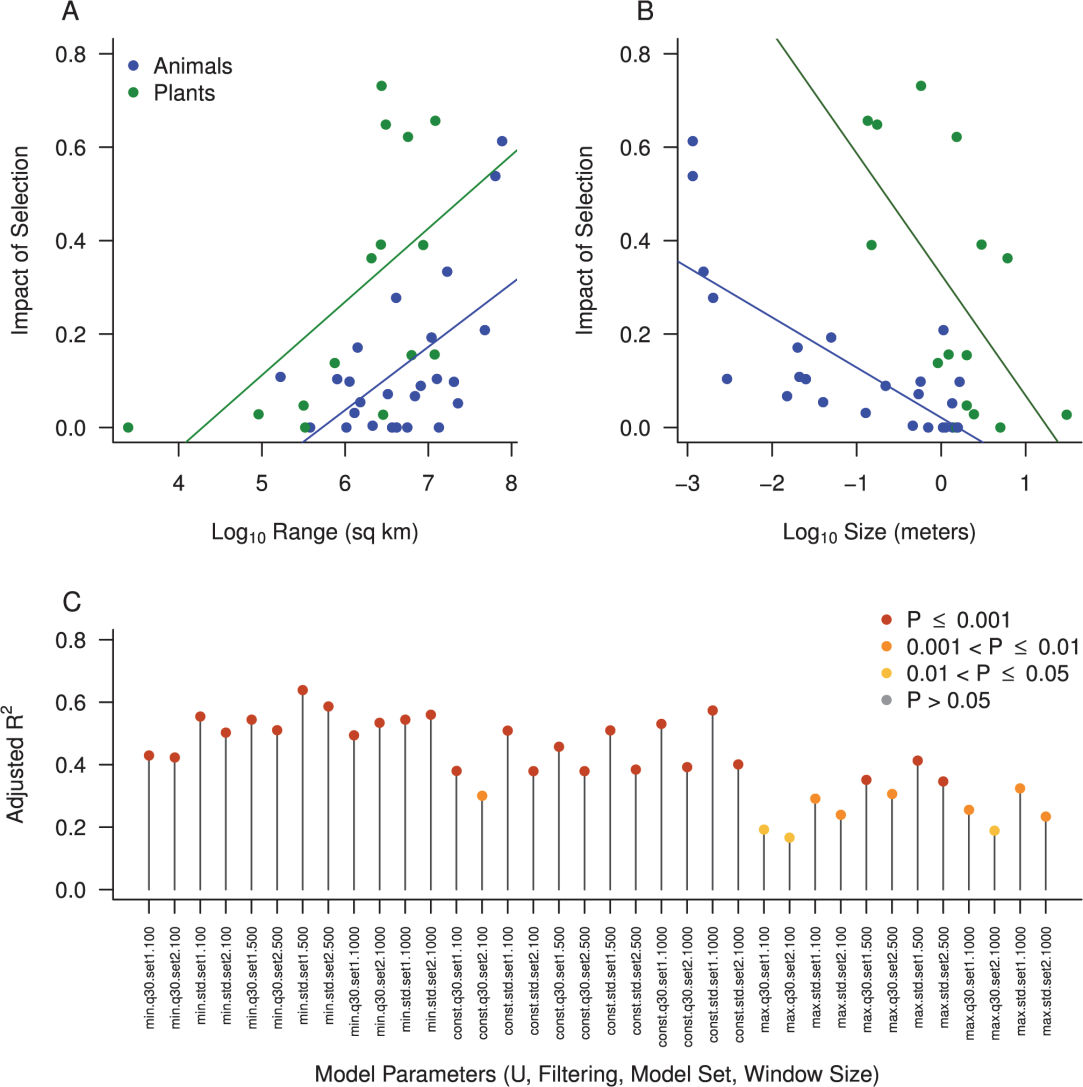
Proxies for census size are correlated with the estimated impact of selection on neutral diversity. For each species, we obtained estimates of size (in meters) and range (in square kilometers), and used those as predictors in a linear model with a measure of the impact of selection on neutral diversity as the response (see main text and Table 2 for full model information). Both range (part A) and size (part B) are significant predictors of the impact of selection on neutral diversity in the expected directions. Points are colored by kingdom (blue = animals, green = plants), and regression lines estimated independently for plants and animals are shown. Raw data underlying this figure can be found in S2 Data. C) Robustness of our model fit. We tested our main model (see text and Table 2) across a wide range of different analysis options, including different filtering options, different window sizes, and different population genetic parameters. Each point represents the adjusted R^2^ of the full model for one set of parameter values, colored according to *p*-value. R code to replicate this analysis is available at: https://github.com/tsackton/linked-selection/blob/master/final_analysis/linear_models.R.

More generally, we tested the hypothesis that Nc is positively correlated with the impact of selection by fitting a linear model that includes body size, geographic range, kingdom, and the significant interactions among them as predictors, and uses the impact of selection estimated from our coalescent model as the response variable (Table 2; Fig. 3). Both size and range are significant predictors of the impact of selection in the expected directions (Table 2; log10(size): coefficient = −0.092, *p* = 0.0005; log10(range): coefficient = 0.112, *p* = 0.0002), and model as a whole explains 63.88% of the variation in impact of selection across species (Table 2; overall *p* = 3.518 x 10^−8^). This is clear evidence that more variation is removed by linked selection from the genomes of species with smaller body size and larger ranges than from the genomes of species with larger body size and smaller ranges.

**Table 2:** Linear model fit for the main model.

	Estimate	Std. Error	*t* value	Pr(>|t|)
(Intercept)	−0.71408	0.18282	−3.906	0.000410
Log_10_ (range)	0.11239	0.02742	4.100	0.000234
Log_10_ (size)	−0.09229	0.02414	−3.823	0.000520
Kingdom (0 = animal, 1 = plant)	0.34567	0.05091	6.789	7.16 × 10^−8^
Log_10_ (size): Kingdom	−0.12337	0.06075	−2.031	0.049935

Overall F-statistic: 18.24 on 4 and 35 DF, *p*-value: 3.518 × 10^−8^, adjusted R^2^: 0.6388

A number of confounding factors could potentially influence our conclusions, including variation in map or assembly quality across species, differences in overall recombination rate, and differences in genome size. To test whether these factors can explain our results, we fit a confounder-only model including two measures of genetic map quality (density of useable markers and proportion of total markers scored as useable); two measures of assembly quality (proportion of assembly that is not gaps and proportion of total assembly assembled into chromosomes); overall recombination rate; and genome size. We then compare this confounder-only model to a model that includes all confounding parameters and, in addition, includes our population size proxies (kingdom, size, and range). The model with proxies for Nc both explains substantially more total variation in impact of selection (adjusted R^2^ of 0.6359 compared to 0.3388 for the confounder-only model) and is a significantly better fit to the data (F = 7.7322, df = 4, *p* = 0.0002).

In order to ensure that variable sampling of chromosomes is not a source of bias (given that the number of chromosomes sampled ranges from a minimum of 2 to a maximum of 517; S1 Table), we tested whether sampling depth is correlated with either size or range. In neither case do we find a correlation (size versus sampling depth: Kendall’s τ = 0.022, *p* = 0.84; range versus sampling depth: Kendall’s τ = 0.044, *p* = 0.699). We also find no evidence that species with only two chromosomes sampled are atypical with respect to range (Wilcoxon Rank Sum Test, *p* = 0.944) or size (Wilcoxon Rank Sum Test, *p* = 0.423). Finally, we find no evidence that mean depth per individual is correlated with either size (Kendall's τ = −0.044, *p* = 0.683) or range (Kendall's τ = −0.02, *p* = 0.862). Taken together, these results strongly suggest that the variable sampling across species, both in terms of sequencing depth and in terms of number of chromosomes sequenced, does not bias our conclusions.

To get a lower bound on the proportion of variation in impact of selection explained by our parameters of interest (range, size, kingdom, and the kingdom–size interaction), we fit a linear model with these parameters as predictors and the residuals of the confounder-only model as the response variable (S4 Table, S5 Table). This is a conservative test, as genome size is strongly correlated with body size (Kendall's τ = 0.296, *p* = 0.007 in our dataset). Nonetheless, our proxies for Nc explain 34.05% of the remaining variation in impact of selection after accounting for all confounding parameters (overall model *p* = 0.0008, S4 Table), and 47.36% of the variation after accounting for all confounding parameters except genome size (overall model *p* = 2.042 x 10^−5^, S5 Table).

For five species, our polymorphism data included individuals from domesticated populations, which could potentially affect our conclusions if selection has a different signature during domestication events than it leaves in natural populations. However, removing these five species has virtually no impact on our model fit (overall adjusted R^2^ = 0.6281, overall *p* = 6.094 x 10^−7^, S6 Table), suggesting that their inclusion has not biased our results. Additionally, we obtain similar results if we fit our model (excluding the kingdom term and its interaction with size) to animals and plants independently (S7 Table, S8 Table). Finally, varying the filtering criteria, window size, assumed deleterious mutation rate (U), or population genetic modeling approach produces nearly identical results (Fig. 3C), implying our primary conclusion is robust to a wide range of analysis choices. Taken together, our analysis demonstrates that the central pattern—natural selection reduces neutral diversity more strongly in species with large Nc than in species with small Nc—is consistently observed with both nonparametric model free approaches (Fig. 2; S3 Table) and with explicit population genetic models (Fig. 3A,B, Table 2) across a wide range of possible analysis and filtering choices (Fig. 3C, S4–S8 Tables).

If the process of recombination is itself mutagenic, neutral processes could produce a correlation between recombination and polymorphism [21,25,27]. However, no or very weak correlations between divergence and recombination have been found in most species that have been closely studied [21,25] (reviewed in [27]). Moreover, for those species in which a positive correlation between divergence and polymorphism has been found (e.g., [45,46]), it is likely at least partially the result of linked selection acting on polymorphisms present in the ancestral population [27,32]. Furthermore, the two species that showed the strongest correlation between polymorphism and recombination (partial τ = 0.5196 for *D*. *melanogaster*, partial τ = 0.4637 for *Drosophila pseudoobscura*) have no such correlation between recombination rate and divergence either on broad scales [21] or fine scales [25]. Finally, many authors have found strong evidence that recombination is not mutagenic in a number of other animal species (e.g., [28,47,48]), and it therefore appears a general consensus has emerged that recombination-associated mutagenesis is unlikely to influence the overall patterns we report in this work [27].

### Species with Small Census Sizes Show Stronger Evidence for Neutrality

As an alternative approach to estimating the impact of natural selection on linked neutral diversity, we considered whether our proxies for Nc correlate with the strength of evidence that selection shapes patterns of neutral diversity, derived from our population genetic modeling approach. To do this, we focus on the relative likelihoods (Akaike weights) of four models: the BGS+HH model, the BGS-only model, the HH-only model, and the neutral model. These relative likelihoods can be interpreted as the probability that a particular model is the best model according to Akaike Information Criteria (AIC), given the set of models tested and the underlying data.

**Fig 4:**
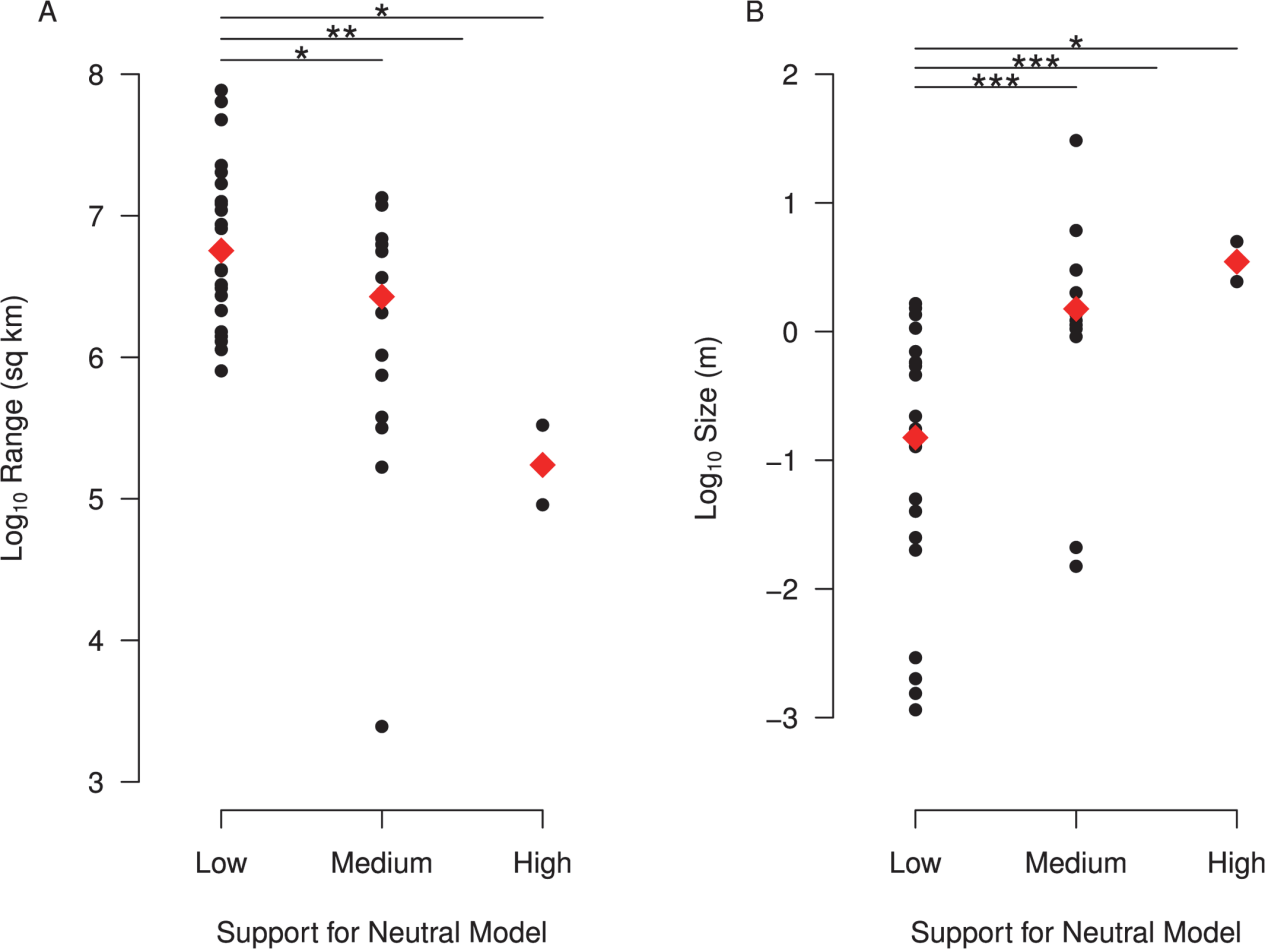
Species with little evidence for selection have smaller census sizes. For each species, we estimated the relative likelihood of a purely neutral model (no impact of selection on neutral diversity), based on AIC values for neutral and selection models (see methods for details) and categorized species based on support for neutrality (low = relative likelihood < 0.05, medium = relative likelihood ≥ 0.05 but < 0.9, high = relative likelihood ≥ 0.9). Species with more support for neutrality have smaller ranges (part A) and larger body size (part B). *p*-values for comparisons (indicated by lines at the top of each panel) of low versus high; low versus medium; and low versus medium and high combined are based on Wilcoxon Rank Sum tests (*** *p* < 0.001, ** *p* < 0.01, * *p* < 0.05). Raw data underlying this figure can be found in S2 Data.

We initially focus on the relative likelihood of the support for a purely neutral model. Species with weak or no support for neutrality (relative likelihood of the neutral model < 0.05) have significantly larger ranges (*p* = 0.006, Wilcoxon Rank Sum Test, Fig. 4A) and significantly smaller sizes (*p* = 0.0001, Wilcoxon Rank Sum Test, Fig. 4B) than species with moderate (relative likelihood of neutral model ≥ 0.05 and < 0.90) or strong (relative likelihood of neutral model ≥ 0.90) support. This pattern also holds if we compare the species with strong support for neutrality or species with moderate support for neutrality individually to species with weak or no support (moderate versus weak: *p* = 0.0005 for size and 0.02 for range; strong versus weak: *p* = 0.02 for size and 0.02 for range, all *p*-values from Wilcoxcon Rank Sum Tests). This suggests that the evidence for non-neutral processes (BGS and/or HH) is significantly stronger in species with larger ranges and/or smaller sizes, consistent with our results above and with the hypothesis that natural selection explains Lewontin's paradox.

### Hitchhiking Appears More Prevalent in Large Nc Species

Given the extensive debate on the relative importance of HH versus BGS in shaping patterns of diversity across the genome [17,21], we also attempt to disentangle the relative roles of these two processes in reducing neutral diversity. This is potentially relevant to the resolution of Lewontin's paradox, as models of frequent, recurrent HH (i.e., genetic draft [7]) demonstrate that recurrent HH can remove the dependence of neutral diversity on population size entirely. Thus, evidence that HH specifically is more likely to occur in species with large census sizes would be compelling evidence for a role of selection in resolving the discrepancy between population sizes and neutral diversity. However, it is crucial to note that our test does not take into account features, such as patterns of polymorphism around amino acid fixations [23,49], that are particularly powerful for distinguishing HH and BGS, and thus suffers from many of the limitations of previous work relying purely on patterns of neutral diversity across the genome (e.g., [26,28,40,41]).

With that caveat, we begin by noting that, consistent with recent work in *Drosophila [* 49,50] and other organisms [26,28,48], background selection is ubiquitous. Either the BGS-only model or the BGS+HH model has at least some support (relative likelihood ≥ 0.05) for 95% (38 of 40) of the species we analyzed, and for 90% (36 of 40) of species one of the BGS-containing models was the best fit, as measured by AIC. Thus, it seems clear that, in most cases, BGS is a more appropriate null model for tests of natural selection than strict neutrality.

To test whether species with moderate (relative likelihood of HH or BGS+HH ≥ 0.05 and < 0.9) or strong (relative likelihood of HH or BGS+HH ≥ 0.9) evidence for HH differ from species with little or no evidence for HH (relative likelihood of HH or BGS+HH < 0.05), we examined our proxies for Nc among these evidence classes. Species with moderate or strong evidence for HH have significantly larger ranges than species with weak or no evidence for HH (*p* = 0.03, Wilcoxon Rank Sum Test, median range (weak) = 2,681,693 sq km, median range (moderate/strong) = 5,592,037 sq km), and these species tend to have smaller sizes as well (*p* = 0.15, Wilcoxon Rank Sum Test, median size (weak) = 0.91 m, median size (moderate/strong) = 0.54 m).

As a second test of this pattern, we compared whether the relative likelihood of HH was greater for species estimated to have particularly high Nc compared to species estimated to have particularly low Nc. We define the high-Nc class as those species with ranges greater than the median range, and sizes below the median size, and we define the low-Nc class as those species with ranges below the median range and sizes above the median size. The relative likelihood of HH models is greater for species in the high-Nc class than the low-Nc class (*p* = 0.023, Wilcoxon Rank Sum Test), and the proportion of species with moderate or strong evidence for HH (either alone or in combination with BGS) is higher in the high-Nc class than the low-Nc class (4/10 in high-Nc class, 0/10 in low-Nc class, *p* = 0.086, Fisher's Exact Test).

Despite the fact that our test is unlikely to have substantial power to distinguish BGS and HH models, we suggest that these results imply that HH in particular is a stronger force shaping genomic diversity in species with large Nc, while BGS appears to be much more pervasive. The observation that pervasive HH may predominantly occur in species with large Nc suggests that genetic draft may play a substantial role in limiting neutral diversity among the species with the largest population sizes. More data on species with very large Nc, and the application of tests specifically designed to detect HH to a wider taxonomic range, will be necessary to fully disentangle the relative roles of HH and BGS in shaping levels of neutral diversity.

## Discussion

On the strength of early allozyme polymorphism data, Lewontin [6] observed that in contrast with theoretical predictions of the neutral theory [51–53], the range of neutral genetic variation among species is substantially smaller than the range of Nc among species. Because both positive selection via HH and negative selection via BGS purge linked neutral mutations, the operation of natural selection affects patterns of neutral genetic variation at linked sites across the genome. Although many authors have suggested that natural selection may play a role in truncating the distribution of genetic variation and may play a greater role than neutral genetic drift in shaping patterns of neutral nucleotide polymorphism [7,8,14,15], few empirical tests of this hypothesis have been proposed or conducted. Here, we show that species with larger Nc display a stronger correlation between neutral polymorphism and recombination rate, and that natural selection removes disproportionately more linked neutral variation from species with larger populations. This indicates that natural selection plays a disproportionately large role in shaping patterns of polymorphism in the genome of species with large Nc.

One important consideration when interpreting our results is that cryptic population structure can influence patterns of variation across the genome in a way that obscures the effects of selection. In the extreme case, where populations do not exchange any migrants for an extended period of time, genetic divergence is expected to accumulate at equivalent rates across the genome and would obscure the effects of linked selection. Elucidating the complex relationship between population structure and patterns of natural selection is an important and longstanding question in population genetics (for recent work see [54,55]). Nonetheless, especially given the scope of our analysis, it is not feasible to simultaneously estimate the effects of linked selection and population structure, and there are many reasons to believe that the results presented here will be robust to potential cryptic population structure.

So long as the population subdivision is not especially ancient (in the timescale of coalescence, on the order of Ne generations), a correlation between recombination and polymorphism is expected to remain due to the effects of selection on linked sites in the ancestral population [27,32]. Additionally, if migration is sufficiently common, it is reasonable to treat data derived from samples from separate localities as a single population [56]. One straightforward assumption is that species with larger geographic ranges will have greater opportunity on average to accumulate cryptic population structure than species with small ranges, which would imply we should preferentially underestimate the effects of linked selection in species with larger ranges. If population structure is a primary determinant of patterns of nucleotide diversity in taxa that we studied, we could reasonably expect a negative correlation between species range and the effects of selection on linked sites. Given that we instead obtain the opposite effect—one consistent with the effect of selection on linked neutral sites—it is reasonable to conclude that cryptic population structure has not drastically influenced the basic results presented herein.

Understanding the proximate and ultimate factors that affect the distribution of genetic variation in the genome is a central and enduring goal of population genetics and it carries important implications for a number of evolutionary processes. One implication of this work is that in species with large Nc, such as *D*. *melanogaster*, selection plays a dominant role in shaping the distribution of molecular variation in the genome. Among other things, this can affect the interpretation of demographic inferences because it indicates that even putatively neutral variants are affected by natural selection at linked sites. Furthermore, to whatever degree standing functional variation is also affected by selection on linked sites (e.g., [40]), local recombination rate in organisms with large Nc may also predict what regions of the genome will contribute the greatest adaptive responses when a population is subjected to novel selective pressures.

More broadly, this work provides direct empirical evidence that the standard neutral theory may be violated across a wide range of species. Indeed, it is clear from this work that in many taxa, natural selection plays a dominant role in shaping patterns of neutral molecular variation in the genome. It is therefore essential to consider selective processes when studying the distribution of genetic diversity within and between species. Incorporating selection into standard population genetic models of evolution will be a central and important challenge for evolutionary geneticists going forward.

## Materials and Methods

### 1. Data Sources and Curation

Reference genome versions, annotation versions, map references, and other basic information about the genetic and genomic data for species we included in our analysis is summarized in S1 Table and S2 Table, and described in more detail below.

#### Reference genomes

To identify suitable species for our analysis, we started from the list of genome projects available at Genomes OnLine Database (GOLD; http://www.genomesonline.org/) and National Center for Biotechnology Information (NCBI; ftp://ftp.ncbi.nlm.nih.gov/genomes/GENOME_REPORTS/), both accessed 6 October 2013. We removed all noneukaryotes from both sets. We then further filtered the GOLD set to remove all projects where status was not either “draft” or “complete” and where project type was not “Whole Genome Sequencing,” and the NCBI set to keep only all projects with MB > 0 and status equal to “scaffold,” “contigs,” or “chromosomes.” Finally, we merged both lists, removed duplicate species, and removed all species without an obligate sexual lifestyle. We required species have an obligatory sexual portion of their lifecycle to ensure that some amount of recombination can be expected in natural populations.

Next, we manually checked the quality of the genome assembly of each species remaining on our list by inspection of assembly reports available from NCBI, Ensembl, Phytozome, or species-specific databases. Any species without chromosome-scale assemblies was removed, as was any species without an available annotation of coding sequence. In two cases (*Heliconius melpomene* and *Gasterosteus aculeatus*), chromosome scale assemblies were available but annotations were only available for the scaffold-level (or a previous, lower-quality chromosome-level) assembly. In these cases, we updated the coordinates of the coding sequence annotations using custom Perl scripts (available from the GitHub page associated with this manuscript: see the data accessibility section for details on how to obtain source code and data).

#### Polymorphism

We required that each species be represented by random-shearing Illumina short read sequence data for at least two chromosomes derived from unrelated individuals within the same population. For four species (*Bos taurus*, *Lepisosteus oculatus*, *Prunus persica*, and *Papio anubis*) we used a single outbred diploid individual. If samples were intentionally inbred or if the species is known to engage in frequent self-fertilization in natural populations, we required data from at least two separate individuals. The number of individuals included and the number of unrelated chromosomes (ploidy in shorthand) of the sequenced individuals are reported in S1 Table. For six species, we used polymorphism data from a very closely related taxon to the species that was sequenced to produce the reference genome (S1 Table). In particular, we attempted to avoid using polymorphism data from domesticated species where possible; in many cases, we were able to use polymorphism data from wild ancestors or close relatives of domesticated plants and animals. Nonetheless, for five species *(Gallus gallus*, *Bos taurus*, *Melagris gallopavo*, *Setaria italica*, and *Citrus reticulata*), we could not identify suitable data from wild populations, and we instead elected to use polymorphism data from heritage breeds and strains (S1 Table).

#### Genetic maps

We required that each species have available a pedigree-based genetic map, generated from markers that could be mapped to the reference genome by either ePCR or Basic Local Alignment Search Tool (BLAST), and with an average intermarker spacing (after filtering unmapped and mismapped markers; see below) of no more than 10 cM. For species with recombination in both sexes, we used sex-averaged genetic distances where possible, although in two cases (*Anopheles gambiae* and *Bos taurus*), maps were only available for a single sex and so for those species we use a single-sex map by necessity. For species with recombination in only one sex (e.g., *Drosophila*), we corrected genetic distances to represent a sex-averaged value by dividing estimated recombination rates in the sex with recombination by two. In nine cases where genetic maps from the same species as the polymorphism data were unavailable or of insufficient quality (*Zea mays*, *Prunus persica*, *Papio anubis*, *Oryza sativa*, *Ovis aries*, *Mus musculus*, *Equus caballus*, *Canis lupus familiaris*, and *Citrus clementina*), we used genetic maps from a closely related taxon, typically the reference genome species (see S1 Table for full details).

#### Range and size information

While ideally we would obtain estimates of actual census population sizes, even moderately accurate estimates are rarely available. As an alternative, we used species range and individual size as proxies for census population size. To determine range, we used occurrence data available from GBIF (http://www.gbif.org/) or published literature (when no occurrence data was available in GBIF) to estimate species distributions as follows. First, for each species, we obtain and then filter all occurrence data stored at GBIF. In general, we filter to require a known source (basis of observation) and exclude fossil records; we also filter to remove clearly erroneous points, such as those well outside the known species range (often arising, e.g., from transposition of longitude and latitude during data entry in museum records) or those falling in oceans for terrestrial organisms and vice versa. Specific filtering steps for each species are documented in the associated R code (available at GitHub). After filtering, we fit an alpha-hull [57] to estimate the species range, which we then filter to remove area overlapping ocean for terrestrial species and overlapping land for oceanic species, and then convert to area by projecting from GPS (WGS84) coordinates to a cylindrical equal area projection using the spTransform function in the R package rgdal (http://cran.r-project.org/web/packages/rgdal/). R scripts to replicate our analysis are provided at the GitHub page associated with this manuscript.

We recognize that GBIF occurrence data reflects current range and does not account for historical range; however, as accurate long-term historical ranges are not known for most species we are limited to using the data that is available. We also note that for the five domesticated species, plus humans, occurrence data is not of much use for estimating range; in these cases we have attempted to approximate either the historical range of the species (humans, turkey, and clementine) or the current range of the heritage breeds the polymorphism sample is obtained from (chicken, cow, and millet).

In order to account for variation in population density across species, we also use individual size as a second proxy for Nc. Body size has been validated as a proxy for local population density across a wide variety of systems and taxa (e.g., [42–44]) and is readily available from common databases such as the Encyclopedia of Life and Animal Diversity Web (S9 Table). While it would be ideal to obtain quantitative estimates of population densities (e.g., by using extensive mark-recapture methods [58]), for the majority of species we studied reliable direct estimates of population densities are not available.

### 2. Polymorphism Pipeline

#### Alignment and genotyping pipeline

We acquired short read data from the NCBI short read trace archive. All accession numbers for short read data used in this analysis are listed in S10 Table. We aligned these data to their respective reference genomes (reference genome versions and relevant citations are listed in S1 Table). For libraries prepared from genomic DNA, we used bwa v0.7.4 [59] with default options. For libraries prepared from RNA, we aligned reads initially using tophat2 v2.0.7 [60] with default options, except we specified “-no-novel-juncs” and “—no-coverage-search” and gave tophat2 a general feature format (GFF) file (version indicated in S1 Table) to speed up alignment. For both DNA and RNA, we then realigned reads that failed to align confidently using Stampy v1.0.21 [61] with default options. After this, putative polymerase chain reaction (PCR) duplicates were removed from both RNA and DNA based libraries using the “MarkDuplicates” function in Picard v1.98 (http://broadinstitute.github.io/picard/). For DNA libraries, we next use the “IndelRealigner” tool in the Genome Analysis Toolkit (GATK) v2.4–3 [62] to realign reads surrounding likely indel polymorphisms. These GATK and Picard functions were run using default command line options.

We genotyped all samples using the GATK v2.4–3 [62]. If samples were intentionally inbred, or if the species is known to primarily reproduce through self-fertilization in natural populations, we used the “-ploidy” option to set the expected number of chromosomes per individual to 1 (see S1 Table for ploidy settings used for each species). We then extracted polymorphism data from four-fold degenerate synonymous sites. While there is mounting evidence that these sites are not evolving under strictly neutral processes (e.g., [63,64]), four-fold degenerate sites are a widely accepted approximation for neutral markers in the genome, and importantly, these sites are available in both RNA and DNA sequencing efforts.

We sought to exclude low confidence sites by filtering our genotype data through several basic criteria. First, we required that every 4-fold degenerate site have a minimum phred-scaled probability of 20 that there is a segregating site within the sample. To ensure robustness of our results, we also applied a more stringent Q30 genotype quality filter and performed otherwise identical analyses using these data. Second, for every 4-fold degenerate site, we computed the mean depth for each sample. We then required each sample have at least half as many reads as the mean depth at a site for that position to be included in the analysis. For variable sites, we further required that phred-scaled strand bias be below 40. This quantity is based on an exact test for how often alternate alleles are called by reads aligned to the + versus the − strand of the reference genome; a large bias might be expected if, for example, a nearby transposable element insertion relative to the reference genome influenced read alignments on one strand and would make the genotypes at that site unreliable. We further required that the absolute value for the Z-score associated with the read position rank sum, the mapping quality rank sum, and the base quality rank sum be above four. These statistics quantify how biased the reference allele alignments relative to those of nonreference alleles for the relevant filters. For example, the first filter—read position rank sum—quantifies whether nonreference alleles are generally found further forward or backward in a short read. This filter may also reflect errors due to systematic differences in alignments of nonreference allele bearing reads (e.g., due to indels on one of the chromosomes present in an individual). See the GATK [62] documentation for in-depth descriptions of the relevant filters used. We applied these criteria to both DNA and RNA based libraries. Summaries of sites aligned and filtered for each genome are available in S11 Table, and a schematic of our pipeline is presented in S1 Fig.

#### Homo sapiens

Rather than recompute variant calls, for the human data, we obtained Variant Call Format (VCF) files for the Yoruban population from [38]. We elected to do this because these data are exceedingly well curated and the size of the human variation raw data presents a practical computational challenge. The VCF file was treated as described below in all case.

#### Estimating genetic diversity in genomic windows

From these filtered files, we computed genetic diversity as π, the average number of pairwise differences [65], at 4-fold degenerate sites in nonoverlapping windows of 100 kb, 500 kb, or 1,000 kb. In all cases, we excluded windows from our analysis with fewer than 500 sequenced 4-fold degenerate sites. We also exclude all windows on sex chromosomes, in order to avoid complicating effects of hemizygosity on patterns of polymorphism.

### 3. Recombination Rate Estimation Pipeline

Our approach to estimating recombination rates is to first obtain sequence information and genetic map positions for markers from the literature, map markers to the genome sequence where necessary, filter duplicate and incongruent markers, and finally estimate recombination rates from the relationship between physical position and genetic position. Specific details of map construction for each species are described in S1 Text.

#### Data curation and mapping markers to the reference genome

We used three basic approaches to link markers from genetic maps to sequence coordinates. In some cases, sequence coordinates are available from the literature, in which case we use previously published values (in some cases updated to the latest version of the genome reference). For cases where primer information (but not full sequence information) is available, we used ePCR [66] with options -g1 -n2 -d50–500 and keeping all successful mappings, except where noted. For cases where locus sequence information is available, we used blastn with an e-value cutoff of 1 × 10^−8^ and retained the top eight hits for each marker, except where noted. In both cases, we only retain positions where the sequence chromosome and the genetic map chromosome are identical. Specific curation and data cleaning steps for individual species are summarized in S12 Table and described in more detail in S1 Text.

#### Removal of incorrectly ordered or duplicated markers

For most species, the genetic position and physical position of markers along a chromosome are not completely congruently ordered. That is, physical position is typically not strictly monotonically increasing with genetic position. Incongruent markers can arise from incorrect genome assemblies, errors in map construction, or sequence rearrangements between the reference genome and the mapping population.

For consistency, we assume that the reference genome is correctly assembled, and we correct the order and orientation of genetic maps to be consistent with the sequence assembly. To remove incongruent markers, we find the longest common subsequence (LCS) of ranked genetic and physical positions and define as incongruent all markers that are not part of the LCS. After removing incongruent markers, we filtered each map to retain only the single-most congruent mapping position for markers with multiple possible genomic locations. Functions to perform this analysis in R are available at the GitHub page associated with this manuscript.

#### Masking low quality map regions

To improve the quality of our recombination rate estimation, we designed a masking filter to exclude regions of chromosomes where the fit between the genetic map and the physical position of markers is particularly poor, defined as a run of five bad markers (for chromosomes with at least 25 markers), or a run of 0.2 times the number of markers on the chromosome, rounded up, bad markers (for chromosomes with at fewer than 25 markers). We also completely mask any chromosome with fewer than five markers in total. The final map quality and various filtering results are summarized in S13 Table.

#### Recombination rate estimation

Our basic approach to recombination rate estimation is to fit a continuous function to the Marey map relating genetic position and physical position for each chromosome. We use two different approaches that result in different degrees of smoothing: a polynomial fit and a linear B-spline fit. In both cases, we start by optimizing the polynomial degree or spline degrees of freedom using a custom R function that maximizes the AIC for the model fit. For the polynomial fit, we optimize between degree 1 and degree max(3, min(20, # markers / 3)). For the B-spline fit, we optimize degrees of freedom between 1 and min(100, max(2, #markers/2)). In each case, we retain the value with the highest AIC. To compute recombination rates in cM/Mb, we then take the derivative of the fitted function, evaluated at the midpoint of each window. For additional smoothing, we set all values of recombination estimated below zero to zero, and all values above the 99th percentile to the 99th percentile. While the two estimates tend to be highly correlated with each other, the polynomial fit appears to perform better for low quality maps, and the B-spline fit for high quality maps. Therefore, unless otherwise noted, we use the polynomial estimates of recombination rate for maps with intermarker spacing of greater than 2 cM, and the B-spline estimates for maps with intermarker spacing less than or equal to 2 cM. All estimation was done in R; code is available at the GitHub page associated with this manuscript.

#### Partial correlations between recombination rate and genetic diversity

To estimate the strength of the association between recombination rate and genetic diversity, we use partial correlations that account for variation in coding sequence density across the genome. In many species [26,40], recombination rate and/or neutral diversity is correlated with gene density, and thus we need to account for this confounding variable in our analysis. We do this using partial correlations, implemented with the ppcor package in R.

First, we estimate coding sequence density in each window as the fraction of each window represented by exonic sites, extracted from the same GFF files for each species used to compute four-fold degenerate sites. We then estimate Kendall's τ between recombination rate and genetic diversity for each window after correcting for coding sequence density.

### 4. Modeling the Joint Effects of Background Selection and Hitchhiking on Neutral Diversity

We begin with the very general selective sweep model derived by Coop and Ralph [41], which captures a broad variety of HH dynamics. To include the effects of BGS, we rely on the fact that to a first approximation, BGS can be thought of as reducing the effective population size and therefore increasing the rate of coalescence. This effect can be incorporated by a relatively simple modification to equation 16 of [41]. Specifically, we scale N by a BGS parameter, exp(-G), in equation 16, which then leads to a new expectation of average pairwise genetic diversity (π):

$$E\left[\pi \right]=\frac{\theta }{1/exp\left(-G\right)+\alpha /rbp}$$ (1)

where α = 2N * Vbp * J2,2 (per [41]) and rbp is the recombination rate per base pair. This is very similar to previously published models of the joint effects of background selection and HH (e.g., [39]). To account for variation in the density of targets of selection, we build upon the approach of Rockman et al. [40] and Flowers et al. [26], which derives from the work Hudson, Kaplan, Charlesworth, and others that originally described models of background selection in recombining genomes [17,18]. Specifically, we fit the following model to estimate G for each window i:

$$G_{i}={\text{Σ}}_{k}\frac{U*fd_{i}*sh}{2*\left(sh+P\left|M_{i}-M_{k}\right|\right)*\left(sh+P\left|M_{i}-M_{k+1}\right|\right)}$$ (2)

where U is the total genomic deleterious mutation rate, fd_i_ is the functional density of window i, sh is a compound parameter capturing both dominance and the strength of selection against deleterious mutations, M_k_ and M_i_ are the genetic positions in Morgans of window k and window i, respectively, and P is the index of panmixis, which allows us to account for the effects of selfing. We estimate functional density as the fraction of exonic coding sites in the genome that fall within the window in question. We focus on exonic coding sites as a proxy for targets of selection as they are the only functional measure that is uniformly available for all the species in our study.

Because P, U, and sh are not known, we fit this BGS model with a variety of parameter combinations. As U is generally unknown, and estimating U is difficult in most cases (e.g., [67,68]), we fit our models with three different values: Umin, where we assume U is equal to the mutation rate times the number of exonic protein-coding bases in the genome; Uconst, where we assume that U is equal to one for all species; and Umax where we assume that U is equal the lesser of the mutation rate times fives times the number of exonic protein-coding bases in the genome or the mutation rate times the genome size. Umin and Umax are multiplied by two to convert to diploid estimates. We believe that these estimates of U should roughly span the reasonable range for most species. Umin is likely to underestimate the true deleterious mutation rate as the number of exonic protein-coding bases will typically underestimate the number of evolutionarily conserved bases in a genome. Umax assumes that 20% of conserved bases are exonic coding bases and 80% are noncoding, which we admit is a relatively arbitrary assumption, but likely close to the maximum plausible U.

For P, we assume one for all vertebrates, insects, and obligate outcrossers among plants; 0.04 for highly selfing species, and 0.68 for partial selfers. These estimates correspond to selfing rates of 0%, ∼98%, and ∼50%, respectively. Estimates of selfing are available in S14 Table. For a few species of plants, we were unable to obtain reliable estimates of selfing rate (indicated by NA in S14 Table), and in this case we include all estimates of P in our model selection approach below. For sh, we fit a range of values evenly spaced (on a log scale) between 1e-5 and 0.1. Code to estimate G_i_ was implemented in C++ and is available from the GitHub repository associated with this manuscript.

To incorporate functional density into the HH component of the model, we make the simplifying assumption that sweeps targeting selected sites outside a window will have little effect on neutral diversity within a window, and that sweeps occur uniformly within a window. Under this assumption, we can consider functional density as a scaling factor on the rate of sweeps, Vbp. Specifically, we reparameterize the rate of sweeps, Vbp, as V, the total sweeps per genome, and then consider the fraction of sweeps that occur in a particular window i as V*fd_i_. This results in a simple scaling of α in Equation 1. While we note that this assumption is likely to be violated in practice, it allows us to use the homogeneous sweep model of [41] with different rates of sweeps for each window across the genome. Ultimately, of course, it would be preferable to derive a nonhomogenous sweep model that directly incorporates variation in functional density, but doing so is beyond the scope of this work. However, we believe that our simplifying is likely adequate, as the largest reduction in diversity associated with a sweep is localized to the window containing the swept site (e.g., [41]).

Incorporating the effects of functional density in both BGS and HH, our final model for the expectation of neutral diversity in window i is:

$$E\left[\pi_{i}\right]=\frac{\theta_{neutral}}{1/exp\left(-G_{i}\right)+\alpha *fd_{i}/rbp_{i}}$$ (3)

To obtain an estimate of the effect of selection for each species, we fit this model for estimates of G_i_ derived from different parameter combinations (see above), using the nlsLM() function from the minpack.lm package in R. In addition, we fit three simpler models: a BGS-only model (in which α is 0 and thus the second part of the denominator is 0), an HH-only model (in which G is 0 for all i, and thus the first part of the denominator is 1), and a neutral model in which both G and α are 0, and thus the model predicts that neutral genetic diversity is equal to mean genetic diversity across the genome. Together, we refer to these four models as model set 1. Finally, we fit a second set of models (model set 2) in which we use the same approach to model background selection, but use the homogenous HH model of [41] without modification to allow for variation in functional density across the genome, and thus remove the fd_i_ term from Equation 3.

From each model fit we estimate θ_neutral_ for all four models (full, BGS-only, HH-only, and neutral) and also extract the likelihood of the fit. We then compute the AIC for each parameter combination, extract the fit with the best AIC for each model, and use that AIC to estimate the Akaike weight (relative likelihood) of each model j as

$$REL_{j}=e^{\left(AIC_{min}-AIC_{j}\right)/2}$$ (4)

which we then normalize so that the weights for all four models for a species sum to one. We focus on AIC as it provides a straightforward way to compare non-nested models.

We estimate expected neutral genetic diversity in the absence of selection (θ_neutral_) for each species as the parameter value obtained by the model with the best AIC. We then compute average observed genetic diversity for each species, and report the magnitude of the impact of selection on linked neutral diversity as 1 – (observed / neutral). Values below zero are replaced by zero. This value can be interpreted as the proportion of neutral variation removed by selection acting on linked sites, averaged across the genome.

This modeling approach has some important limitations: in particular, our approach calculates the effects of BGS and HH in windows across the genome instead of per base and we use the parameter *sh* instead of integrating across the distribution of fitness effects (as is done in e.g. [48,50]). Additionally, we do not use information such as locations of amino acid fixations, as is used by [49]. We fully acknowledge that these simplifying assumptions will, to a certain extent, degrade the accuracy of our modeling approach compared to other possible approaches. We argue, however, that these assumptions are necessary for this work: more sophisticated models typically require additional data (e.g., the distribution of fitness effects of new mutations or the location of recent amino acid fixations), or significantly increased computational time (i.e., by computing the effects of background selection at each base instead of in windows). For most of the species we studied, the necessary additional data are not clearly available to fit more complex models, and the increased computational time to fit per-base models would rapidly make our analysis computationally intractable. Thus, we believe that we have made reasonable tradeoffs between modeling complexity, data availability, and taxonomic breadth.

### 5. Linear Models

Our goal is to test whether Nc predicts the degree to which selection shapes patterns of neutral diversity, using log-transformed measures of body size and geographic range as proxies for Nc. However, many other factors could potentially influence our measure of strength of selection, including biological factors such as genome size and average recombination rate; and experimental factors such as map quality and assembly quality. In particular, we might expect to underestimate the strength of selection in species with low-quality assemblies or maps, and we might expect that on average, larger genomes and higher recombination rates would reduce the impact of selection.

In order to account for these parameters that are not directly of interest, we use two approaches. First, we compare a model that includes both our parameters of interest and our parameters not directly of interest to a model that includes only the parameters not directly of interest, in order to test whether our proxies for Nc result in a significantly better fit. Second, we fit our proxies for Nc to the residuals of a linear model including only parameters not directly of interest, in order to determine how much variation proxies for Nc explain after accounting for all the variation that can be explained by genome size, average recombination rate, and quality parameters.

We obtain assembly quality from NCBI, Phytozome, the original genome publication, or compute it directly from fasta files. C-values for plants come from http://data.kew.org/cvalues/, and C-values for animals come from [69]. In all cases, most recent estimates, "prime" estimates, or flow cytometry estimates are preferred; where several seemingly equally good estimates are available, the average is used. In some rare cases, a related species is used instead of the sequenced species if the C-value for the sequenced species is not available. We focus on C-values instead of assembly size as using assembly size as a measure of genome size confounds genome size and assembly quality (lower quality assemblies will be on average less complete and therefore smaller). Assembly parameters and sources are listed in S15 Table. We estimate average recombination rate as the overall map size divided by the size of the genome covered by the map.

In order to determine which interactions among proxies for Nc (size, range, and kingdom) to include, we start with the full model including all interactions and remove all non-significant interactions. After doing so, our model is

$$selectionstrength\sim log_{10}\left(size\right)+log_{10}\left(range\right)+kingdom+log_{10}\left(size\right):kingdom$$ (5)

### 6. Data Accessibility

The data we analyze in this manuscript, and the scripts we used to produce our results, are available as follows. All genomes, polymorphism datasets, and GFF annotation files are publicly available from NCBI or other sources. Genome references and versions are listed in S1 Table, and URLs pointing to the location of genome sequence and GFF annotations are available in S2 Table. Sequence Read Archive (SRA) accessions for polymorphism datasets are listed in S10 Table, and references for polymorphism datasets, where available, are listed in S1 Table. Genetic maps for each species are available from the references listed in S1 Table, or as an R data file available at the GitHub page associated with this manuscript (https://github.com/tsackton/linked-selection). All Perl scripts, R scripts, and C++ code associated with this manuscript are available from GitHub (https://github.com/tsackton/linked-selection), and the function of each piece of code is documented both in comments in the code itself and in the Github README. Programs used for read mapping and genotyping, along with command line parameters, are described in the methods. The GitHub page also provides several intermediate data files, including range and size data for each species, neutral diversity and recombination rate for 100 kb, 500 kb, and 1,000 kb windows across each species, and the final dataset analyzed with the linear models described above.

## Supporting Information

S1 Data Data underlying Fig. 2. (XLS)

S2 Data Data underlying Fig. 3A, Fig. 3B, and Fig. 4. (XLS)

S1 Fig Alignment and SNP calling pipeline. A schematic of the bioinformatic tools used to align DNA-seq and RNA-seq data, call genotypes, and extract putatively neutral sites for analysis. Full command line options of programs used are described in the methods.
(EPS)

S1 Table Details of the species used in this study and the data sources. Includes the genome version used, the details of the genetic map used, and the source of the polymorphism data.
(XLS)

S2 Table Links to online sources for genomes and annotations used in this study. (XLS)

S3 Table Robustness analysis of correlation results. (DOCX)

S4 Table Linear model fit to residuals of nuisance parameter model. (DOCX)

S5 Table Linear model fit to residuals of nuisance parameter model, excluding genome size. (DOCX)

S6 Table Linear model fit after removing domesticated species. (DOCX)

S7 Table Linear model fit to the animals-only data subset. (DOCX)

S8 Table Linear model fit to plants-only data subset. (DOCX)

S9 Table Body size for each species used in this study. (XLS)

S10 Table Accessions for polymorphism data used in this study. (TXT)

S11 Table Filtering statistics and sites aligned for each species. (XLS)

S12 Table Summary of map curation and cleaning steps for each species. (XLS)

S13 Table Summary of genetic map quality for each species. (XLS)

S14 Table Estimates of selfing for plant species and associated references. (XLS)

S15 Table Genome size and other parameters used in linear models. (XLS)

S1 Text Full description of map construction methods. (DOC)

## Abbreviations

AIC: Akaike Information Criteria
BGS: background selection
BLAST: Basic Local Alignment Search Tool
Nc: census population size
GFF: general feature format
GOLD: Genomes OnLine Database
HH: hitchhiking
NCBI: National Center for Biotechnology Information
PCR: polymerase chain reaction
