# Supplementary material for: Natural Selection Constrains Neutral Diversity across A Wide Range of Species
Source: PLoS Biol. 2015 Apr 10;13(4):e1002112. doi: 10.1371/journal.pbio.1002112 (PMC4393120; doi:10.1371/journal.pbio.1002112)
Supplement: S3 Table — (DOCX) [file pbio.1002112.s006.docx]

S3 Table:

Difference in median partial correlations between invertebrates and vertebrates or herbaceous and woody plants, for a variety of window sizes and two different filtering schemes.

| Kingdom | Filtering | Window Size (kb) | Wilcoxon Test P-value | Difference in Medians |
| --- | --- | --- | --- | --- |
| animal | q30 | 100 | 0.0169 | 0.1115 |
| animal | q30 | 1000 | 0.2212 | 0.1319 |
| animal | q30 | 500 | 0.0805 | 0.1158 |
| animal | std | 100 | 0.0169 | 0.1001 |
| animal | std | 1000 | 0.0805 | 0.1139 |
| animal | std | 500 | 0.0476 | 0.0873 |
| plant | q30 | 100 | 0.0188 | 0.0999 |
| plant | q30 | 1000 | 0.0265 | 0.1122 |
| plant | q30 | 500 | 0.0265 | 0.1101 |
| plant | std | 100 | 0.0265 | 0.0997 |
| plant | std | 1000 | 0.0265 | 0.1011 |
| plant | std | 500 | 0.0265 | 0.0979 |
