# Supplementary material for: Natural Selection Constrains Neutral Diversity across A Wide Range of Species
Source: PLoS Biol. 2015 Apr 10;13(4):e1002112. doi: 10.1371/journal.pbio.1002112 (PMC4393120; doi:10.1371/journal.pbio.1002112)
Supplement: S4 Table — (DOCX) [file pbio.1002112.s007.docx]

S4 Table:

Linear model fit for the full residuals model

|  | Estimate | Std. Error | t value | Pr(>\|t\|) |
| --- | --- | --- | --- | --- |
| (Intercept) | -0.564069 | 0.184768 | -3.053 | 0.00431 |
| Log_10_ (range) | 0.081897 | 0.027708 | 2.956 | 0.00555 |
| Log_10_ (size) | -0.003324 | 0.024399 | -0.136 | 0.89242 |
| Kingdom (0=animal, 1=plant) | 0.110979 | 0.051457 | 2.157 | 0.03798 |
| Log_10_ (size) : Kingdom | -0.176618 | 0.061397 | -2.877 | 0.00680 |

Overall F-statistic: 6.035 on 4 and 35 DF, p-value: 0.0008399, adjusted R-squared: 0.3405
