# Supplementary material for: Natural Selection Constrains Neutral Diversity across A Wide Range of Species
Source: PLoS Biol. 2015 Apr 10;13(4):e1002112. doi: 10.1371/journal.pbio.1002112 (PMC4393120; doi:10.1371/journal.pbio.1002112)
Supplement: S5 Table — (DOCX) [file pbio.1002112.s008.docx]

S5 Table:

Linear model fit for the residuals model without genome size

|  | Estimate | Std. Error | t value | Pr(>\|t\|) |
| --- | --- | --- | --- | --- |
| (Intercept) | -0.65723 | 0.19840 | -3.313 | 0.00216 |
| Log_10_ (range) | 0.08105 | 0.02975 | 2.724 | 0.00999 |
| Log_10_ (size) | -0.06851 | 0.02620 | -2.615 | 0.01307 |
| Kingdom (0=animal, 1=plant) | 0.26825 | 0.05525 | 4.855 | 2.49e-05 |
| Log_10_ (size) : Kingdom | -0.13225 | 0.06593 | -2.006 | 0.05263 |

Overall F-statistic: 9.773 on 4 and 35 DF, p-value: 2.042e-05, adjusted R-squared: 0.4736
