# Supplementary material for: Natural Selection Constrains Neutral Diversity across A Wide Range of Species
Source: PLoS Biol. 2015 Apr 10;13(4):e1002112. doi: 10.1371/journal.pbio.1002112 (PMC4393120; doi:10.1371/journal.pbio.1002112)
Supplement: S6 Table — (DOCX) [file pbio.1002112.s009.docx]

S6 Table:

Linear model fit for the main model after excluding domesticated species

|  | Estimate | Std. Error | t value | Pr(>\|t\|) |
| --- | --- | --- | --- | --- |
| (Intercept) | -0.70025 | 0.19278 | -3.632 | 0.001037 |
| Log_10_ (range) | 0.10872 | 0.02823 | 3.851 | 0.000575 |
| Log_10_ (size) | -0.09784 | 0.02513 | -3.893 | 0.000512 |
| Kingdom (0=animal, 1=plant) | 0.34077 | 0.05602 | 6.083 | 1.11e-06 |
| Log_10_ (size) : Kingdom | -0.11200 | 0.06221 | -1.800 | 0.081850 |

Overall F-statistic: 15.36 on 4 and 30 DF, p-value: 6.094e-07, adjusted R-squared: 0.6281
