# Supplementary material for: Natural Selection Constrains Neutral Diversity across A Wide Range of Species
Source: PLoS Biol. 2015 Apr 10;13(4):e1002112. doi: 10.1371/journal.pbio.1002112 (PMC4393120; doi:10.1371/journal.pbio.1002112)
Supplement: S7 Table — (DOCX) [file pbio.1002112.s010.docx]

S7 Table:

Linear model fit for the main model on animals only

|  | Estimate | Std. Error | t value | Pr(>\|t\|) |
| --- | --- | --- | --- | --- |
| (Intercept) | -0.63981 | 0.17368 | -3.684 | 0.001300 |
| Log_10_ (range) | 0.10104 | 0.02632 | 3.839 | 0.000892 |
| Log_10_ (size) | -0.09381 | 0.01593 | -5.890 | 6.3e-06 |

Overall F-statistic: 31.27 on 2 and 22 DF, p-value: 3.709e-07, adjusted R-squared: 0.7161
