# Supplementary material for: Natural Selection Constrains Neutral Diversity across A Wide Range of Species
Source: PLoS Biol. 2015 Apr 10;13(4):e1002112. doi: 10.1371/journal.pbio.1002112 (PMC4393120; doi:10.1371/journal.pbio.1002112)
Supplement: S8 Table — (DOCX) [file pbio.1002112.s011.docx]

S8 Table:

Linear model fit for the main model on plants only

|  | Estimate | Std. Error | t value | Pr(>\|t\|) |
| --- | --- | --- | --- | --- |
| (Intercept) | -0.42824 | 0.34068 | -1.257 | 0.2327 |
| Log_10_ (range) | 0.12206 | 0.05441 | 2.243 | 0.0445 |
| Log_10_ (size) | -0.21188 | 0.08359 | -2.535 | 0.0262 |

Overall F-statistic: 7.667 on 2 and 12 DF, p-value: 0.007158, adjusted R-squared: 0.4878
