# Supplementary material for: Natural Selection Constrains Neutral Diversity across A Wide Range of Species
Source: PLoS Biol. 2015 Apr 10;13(4):e1002112. doi: 10.1371/journal.pbio.1002112 (PMC4393120; doi:10.1371/journal.pbio.1002112)
Supplement: S1 Text — (DOC) [file pbio.1002112.s019.doc]

Anopheles gambiae

Map and markers are derived from <http://www.ncbi.nlm.nih.gov/pmc/articles/PMC1207350/>. Mapped to AgamP3 assembly by ePCR using standard approach described below. Chromosome arms were converted to whole chromosomes for consistency with the map by adding the sequence length of the R arms to the L arms based on VectorBase numbers for chromosome length.

Apis mellifera

Map and marker sequences obtained from the map reference, and mapped to the latest Amel assembly via ePCR.

Arabidopsis thaliana

Map and markers derived from <http://www.ncbi.nlm.nih.gov/pmc/articles/PMC1564425/>. Genomic locations updated to latest assembly (TAIR10) by taking the midpoint of the coordinates for the gene id for each marker (from Supplemental Table 5), based on the mapping data available at <ftp://ftp.arabidopsis.org/home/tair/Maps/mapviewer_data/TAIR9_AGI_gene.data>.

Bombyx mori

Map and markers derived from <http://www.ncbi.nlm.nih.gov/pmc/articles/PMC2395255/>. Mapped to integretedseq assembly from KAIKOBase by ePCR using standard approach described below.

Bos taurus

Map, markers, and positions derived from <http://www.ncbi.nlm.nih.gov/pmc/articles/PMC2680908/>. Converted from Btau4.0 to UMD 3.1 via UCSC LiftOver, with default options. Markers where coordinates were not successfully transferred were removed.

Brachypodim distachyon

Map, markers, and positions derived from supplemental material associated with [http://www.ncbi.nlm.nih.gov/pubmed/21597976](../../../../h)

C. briggsae

Map and positions come from <http://www.ncbi.nlm.nih.gov/pmc/articles/PMC3136444/>. Currently using the F2 map, not the advanced intercross map, as that avoids having to deal with issues of map expansion.

C. elegans

Data downloaded from WormBase at <ftp://ftp.wormbase.org/pub/wormbase/species/c_elegans/annotation/genetic_limits/c_elegans.current.genetic_limits.gff2.gz> on 11/21/2013. Only cloned, absolute position markers are used.

Canis lupus

Map and markers derived from <http://www.genetics.org/content/184/2/595.full>. CanFam2 positions published as supplemental material were converted to CanFam 3.1 positions using UCSC LiftOver, with default options. Markers where coordinates were not successfully transferred were removed.

Capsella rubella

Map, marker, and positions are derived from the genome paper via personal communication.

Citrullus lanatus

Map, markers, and positions come from <http://www.ncbi.nlm.nih.gov/pmc/articles/PMC3256148/>. Scaffold positions were converted to chromosome positions using the supplemental table of scaffold positions on chromosomes from the map reference.

Citrus clementina

Map and markers are derived from the C. clementina map described in <http://www.ncbi.nlm.nih.gov/pmc/articles/PMC3546309/>. Markers were mapped to the reference genome via BLAST as follows:

1) Accessions for each marker were retrieved from the supplemental material in the mapping paper

2) FASTA sequence for each accession was retrieved from Genbank using Batch Entrez agains the GSS, Nucleotide, and EST databases in turn.

3) FASTA sequences were concatenated and blasted against the reference genome with an E-value cutoff of 1e-08 and a max output number of 5 sequences.

4) For each marker query sequence, we kept the single best hit and used the start position of the best HSP as the genomic coordinates of the marker.

Cucumis sativus

Map and markers are derived from <http://www.ncbi.nlm.nih.gov/pubmed/22487099>. Mapped to the reference genome via standard ePCR.

Cynoglossus semilaevis

Map retyped from original figure in reference. Marker accessions obtained from map reference, sequence downloaded from NCBI, and mapped to genome via standard BLAST.

Danio rerio

Map, markers, and positions taken directly from supplemental information associated with <http://www.ncbi.nlm.nih.gov/pmc/articles/PMC3178105/>

Drosophila melanogaster

Map, markers, and positions are derived from Comeron's data (<http://www.ncbi.nlm.nih.gov/pmc/articles/PMC3469467/>), but converting to a cM map (based on assuming c in cM/Mb is constant over each 100kb window, the next midpoint will be c*0.01 cM from the previous midpoint in cM).

Drosophila pseudoobscura

Map, markers, and positions are derived from Noor's data (<http://www.ncbi.nlm.nih.gov/pmc/articles/PMC3496668/>), using the Chromosome 2 map (uncondensed) from the Flagstaff population.

Equus caballus:

The horse map is a sex-averaged map derived from two mapping families described in <http://www.ncbi.nlm.nih.gov/pubmed/16314071>. Data obtained from ArkDB (<http://www.thearkdb.org/arkdbgridqtl/SelectAnalysis.action?speciesid=ARKSPC00000005>), and one error was corrected (Marker NVHEQ232 mislabeled as NVHEW232).

To assign markers to chromosomal locations in the genome sequence, the following procedure was used:

1) All markers that could be identified in the UniSTS map of horse available from <ftp://ftp.ncbi.nih.gov/genomes/MapView/Equus_caballus/sequence/BUILD.2.2/initial_release/> were assigned to the start position of the location indicated in the MapView file. In all cases below the start position (lowest genomic coordinate) was used for consistency.

2) Of the remaining markers, the location was taken from <http://www.ncbi.nlm.nih.gov/pmc/articles/PMC2685479/> for any markers assigned a genomic location in that reference.

3) Remaining markers were mapped by ePCR to the horse genome using standard approaches.

4) For a subset of markers where full sequence accessions could be identified, location was determined by blast against the horse genome using the sequence from Genbank.

Primers and other marker information came from:

1) www.ncbi.nlm.nih.gov/pmc/articles/PMC2587302/ (supplemental table and .md file from Genbank, Equus map viewer)

2) other primer sequences identified from:

http://www.uky.edu/Ag/Horsemap/Maps/LINDGREN.PDF

http://dga.jouy.inra.fr/cgi-bin/lgbc/main.pl?BASE=horse

3) references in ttp://www.ncbi.nlm.nih.gov/pubmed/16314071

Ficedula albicollis

SNP positions extracted from dbSNP records based on published genetic map.

Gallus gallus

Markers, map, and locations were derived from the supplemental material associated with <http://www.ncbi.nlm.nih.gov/pmc/articles/PMC2661806/>. Converted from WASHUC2 (build 2.1) coordinates to Ggal4.0 coordinates via UCSC LiftOver, with default options.

Gasterosteus aculeatus

Markers, map, and locations derived from supplemental material associated with <http://www.ncbi.nlm.nih.gov/pubmed/23601112>.

Glycine max

Markers, map, and locations derived from the Glycine Max Consensus 4.0 map available from Soybase (http://www.soybase.org/dlpages/index.php) and described in [http://naldc.nal.usda.gov/catalog/4252](http://naldc.nal.usda.gov/catalog/42524).

Gossypium raimondii

Diploid D genome map positions (cM) from <http://www.ncbi.nlm.nih.gov/pmc/articles/PMC1470701/>, available as a spreadsheet here: <http://www.plantgenome.uga.edu/cottonmap.htm>. Marker mapping to genome sequence based on BLAST (same settings as ccle) using sequences from the map paper, or the cotton genome database (<http://www.cottongen.org/data/download>) when sequences were not available in map paper.

Heliconius melpomene

Markers, maps, and locations are derived from (<http://www.ncbi.nlm.nih.gov/pmc/articles/PMC3398145/>), with the help of personal communications from John Davey. Chromosomal sequences were assembled from the AGP file available at <http://www.butterflygenome.org/node/4>.

Homo sapiens

Use deCODE genetics (Kong et al 2010) male and female maps, but compute sex-averaged map in R for usage. Using the overall map, not the map broken down by carrier status. Map, markers, and positions available as supplemental data (<http://www.decode.com/addendum/>). Coordinates updated to hg19 via UCSC LiftOver.

Lepisosteus oculatus

Map and marker sequences are from a personal communication. Marker sequences were mapped to the genome via standard BLASTN.

Macaca mulatta (mmul):

The map was assembled from data available at the website of the Southwest Primate Research Center, here: <http://baboon.txbiomedgenetics.org/Rhesus_Results/RhesusMapSummary.php>, based on two papers (<http://www.ncbi.nlm.nih.gov/pubmed/17010566>, [http://www.ncbi.nlm.nih.gov/pubmed/16321502](../../../../h),

and possibly further additional work). Markers downloaded from same source. Positions obtained for each marker by standard ePCR.

Medicago truncatula:

The map is the UMN Young 2006 integrated map, described in part here (<http://www.ncbi.nlm.nih.gov/pmc/articles/PMC1456377/>) and available from the Legume Information Service here: <http://cmap.comparative-legumes.org/cgi-bin/cmap/map_set_info?map_set_acc=MtYoungUMinn2006>.

We obtained marker information (primer sequences) from medicago.org (<http://www.medicago.org/genome/downloads/Mt_genetic_markers_Oct_2009.txt>) and mapped markers to the genome with ePCR as per standard approach.

Meleagris gallopavo

Map, markers, and positions downloaded from the supplement of this paper: <http://www.biomedcentral.com/1471-2164/11/647>

Mus musculus

The map is the new standard map, published here (<http://www.genetics.org/content/182/4/1335.full>), and available to download here (<http://cgd.jax.org/mousemapconverter/>). Coordinates were updated from NCBI Build 37 to GRCm38 coordinates using UCSC LiftOver.

Oryza sativa

The map is derived from the published map described here (<http://www.ncbi.nlm.nih.gov/pubmed/23918062>). To generate the map, we downloaded data and R scripts from <http://www.ricediversity.org/data/> and the supplemental materials of the listed reference and recapitulated the map reconstruction algorithm in R/qtl. The map was confirmed identical to the published version. The coordinates of each marker were converted from MSU 6 to MSU 7 (IRGSP 1.0) using the coordinate conversion tool at gramene.org.

Oryzias latipes

Download mappedGeneticmarkers track from the Medaka browser, described here: <http://medaka3.utgenome.org/~kobayashi/UTGBmedakaHELP/GneticMarkers.html>. File is medaka_version1.0.mapping.xml. Parse markers and genetic map positions, get primer information from SNPs_markers(TUN_series).xls also available from the Genetic Markers section of Medaka browser. Map to genome by ePCR per standard approach. We only used markers and map positions derived from the HdrR-HNI backcross detailed in <http://www.ncbi.nlm.nih.gov/pubmed/17554307> and http://www.ncbi.nlm.nih.gov/pubmed/16226856

Ovis aries

The domestic sheep map is version 4.7 of the SM/IMF map, available from NCBI MapViewer ftp site as 9940.SM4.7.md. This represents the latest version of the sex-averaged map originally described in http://www.ncbi.nlm.nih.gov/pubmed/11435411. Since this is exclusively an STS map, genomic positions for each marker were obtained by cross-referencing the seq_sts.md file also available from NCBI (positions are computed by ePCR at NCBI).

Papio anubis

Map downloaded from <http://baboon.txbiomedgenetics.org/Bab_Polymorphisms/ChromInfoBL.php>, primers downloaded from same site, mapped to genome by ePCR using standard approach. Note: mapping data is from P. hamadryas.

Populus trichocarpa.

Map and some marker information is derived from the supplemental materials of this paper: <http://www.ncbi.nlm.nih.gov/pubmed/19220791>.

Sequence information for probes comes from the published map paper; primer information for SSRs comes from <http://web.ornl.gov/sci/ipgc/ssr_resource.htm>. We made a few assumptions about nomenclature to link the map to the SSR file: the r before the name of some markers is meaningless, and that O = ORPM_, P = PMGC_, and W = WPMS_. Markers ending in (a) or (b) are assumed to be the same thing, and are kept only if they match the genome location.

Mapping of sequence to genome coordinates is via ePCR for primers and BLASTN for probes, using standard approach.

Prunus persica

The genetic map we are using is the T x E map, available (along with several other maps) at <http://www.rosaceae.org/species/prunus_persica/genome_v1.0> along with Peach v1.0 positions for all markers (<http://www.rosaceae.org/sites/default/files/peach_genome/GDR_markers_gbrowse.xls>)

Setaria italica

The map, markers, and positions all come from Supplemental Table 4 of the map reference (<http://www.nature.com/nbt/journal/v30/n6/full/nbt.2196.html>).

Sorghum bicolor

The genetic map is the CIRAD map, available as supplemental information from here (<http://www.ncbi.nlm.nih.gov/pmc/articles/PMC2671505/>). Mapped positions in the genome are obtained from the analysis published here (<http://www.ncbi.nlm.nih.gov/pubmed/21484332>).

Sus scrofa

Direct estimates of recombination rate between 1 Mb bins were obtained from <http://www.ncbi.nlm.nih.gov/pubmed/23152986> and converted to a genetic map in cM for analysis.

Zea mays

We used the NAM map, which is available with markers already mapped from <http://www.panzea.org/db/gateway?file_id=NAM_map_and_genos>.
